# Predictors of nursing home admission in the older population in Belgium: a longitudinal follow-up of health interview survey participants

**DOI:** 10.1186/s12877-022-03496-4

**Published:** 2022-10-20

**Authors:** Finaba Berete, Stefaan Demarest, Rana Charafeddine, Karin De Ridder, Johan Vanoverloop, Herman Van Oyen, Olivier Bruyère, Johan Van der Heyden

**Affiliations:** 1 Department of Epidemiology and public health, Sciensano, Juliette Wytsmanstraat 14, 1050 Brussels, Belgium; 2 Department of Public Health, Epidemiology and Health Economics, University of Liège, Liège, Belgium; 3 Intermutualistic Agency (IMA-AIM), Brussels, Belgium; 4 Department of Public Health and Primary Care, Ghent University, Ghent, Belgium; 5 WHO Collaborating Centre for Public Health aspects of musculoskeletal health and ageing, Division of Public Health, Epidemiology and Health Economics, University of Liège, Liège, Belgium

**Keywords:** Nursing home admission, Institutionalization, Older adults, Predictors, Administrative data, Linkage, Competing risk analysis

## Abstract

**Background:**

This study examines predictors of nursing home admission (NHA) in Belgium in order to contribute to a better planning of the future demand for nursing home (NH) services and health care resources.

**Methods:**

Data derived from the Belgian 2013 health interview survey were linked at individual level with health insurance data (2012 tot 2018). Only community dwelling participants, aged ≥65 years at the time of the survey were included in this study (*n* = 1930). Participants were followed until NHA, death or end of study period, i.e., December 31, 2018. The risk of NHA was calculated using a competing risk analysis.

**Results:**

Over the follow-up period (median 5.29 years), 226 individuals were admitted to a NH and 268 died without admission to a NH. The overall cumulative risk of NHA was 1.4, 5.7 and 13.1% at respectively 1 year, 3 years and end of follow-up period. After multivariable adjustment, higher age, low educational attainment, living alone and use of home care services were significantly associated with a higher risk of NHA. A number of need factors (e.g., history of falls, suffering from urinary incontinence, depression or Alzheimer’s disease) were also significantly associated with a higher risk of NHA. On the contrary, being female, having multimorbidity and increased contacts with health care providers were significantly associated with a decreased risk of NHA. Perceived health and limitations were both significant determinants of NHA, but perceived health was an effect modifier on limitations and vice versa.

**Conclusions:**

Our findings pinpoint important predictors of NHA in older adults, and offer possibilities of prevention to avoid or delay NHA for this population. Practical implications include prevention of falls, management of urinary incontinence at home and appropriate and timely management of limitations, depression and Alzheimer’s disease. Focus should also be on people living alone to provide more timely contacts with health care providers. Further investigation of predictors of NHA should include contextual factors such as the availability of nursing-home beds, hospital beds, physicians and waiting lists for NHA.

**Supplementary Information:**

The online version contains supplementary material available at 10.1186/s12877-022-03496-4.

## Background

The ageing of populations combined with the increase in the prevalence of chronic conditions and the rapid advance in medical technology may lead to an increase of long-term care (LTC) services for older people [1]. In most industrialized countries, the demand for nursing homes (NH) is expected to rise sharply during the coming years [1–4]. According to the demographic projections of the Belgian Federal Planning Bureau in 2019, the share of the population aged 67 and over, which was 16% in 2018, will rise to 20% in 2030 and 23% in 2070 [5]. Therefore, the number of people requiring care either at home or at a NH will increase. Data from the Intermutualistic Agency (IMA) show that in 2020, 5,3% of the population aged 65 years and older resided in a NH [6]. If health policies focus on healthy ageing and the organization of well-functioning and integrated home care, the need for more NH could be reduced.

According to a study conducted by the King Baudouin Foundation, 80% of Belgian older people wish to live at home as long as possible [7]. However, this can only be achieved in case of a suitable family or financial situation, or in the absence of serious medical problems. Otherwise, older people can rely upon a whole chain of care services, from home care through intermediate forms to permanent care. An intermediate form of services consists of day care centers and short-stay centers, while permanent care and support in a residential center is found at the end of the care services chain [8]. Day-care centers are a solution for people who are able to live at home but who do not have someone coming in daily to provide the necessary help and care. A short-stay center offers much of the same care as a permanent center for residential care, but the stay is limited in time (a maximum of 90 days, of which a maximum of 60 days can be consecutive, per year). In most cases short-stay centers are embedded in residential care centers, which then include a number of beds specifically designated for short-stay residents. As to the residential care centers in which older people stay permanently, a distinction should be made between a rest home, on the one hand, and a rest and care home, on the other hand. Only the latter are able to accommodate people with a high level of dependency and who require more extensive care. Most residential homes in Belgium are recognized as being rest and care homes, which means that they include both residents with and without special care needs. To ease reading, both the rest homes and the rest and care homes will be referred to as nursing homes in this paper.

Although living independently at one’s own home within the community is a major objective defining healthy ageing [9], the organization of LTC must take into consideration the balance between community and institutional care, which both have financial costs and societal impacts [9–11]. For a better planning of the future demand for NH services and health care resources, policy makers need to be aware of the predictors of nursing home admission (NHA). Previous studies have identified potential predictors of NHA on the basis of Andersen’s behavioral model of health services use, which considers the use of health services to be a function of an individual’s predisposing, enabling, and need characteristics [2, 3, 12–15]. Predisposing factors include demographics, social structure, and health beliefs. Enabling factors are those influencing an individual’s ability to gain access to health services and include family and community resources. Need factors refer to the functional and health problems that generate the need for health care services [12]. The most relevant are marital status (being single or widowed), higher severity of cognitive impairment and mobility impairment [1], dementia [16, 17], living situations and older age [2, 10, 12, 18–20]. The role of urinary incontinence as predictor of NHA remains controversial. Some studies found that urinary incontinence is a strong predictor of NHA [21, 22], while another study found that urinary incontinence was not an independent predictor of NHA after adjusting for confounders [23].

An important limitation of most previous studies on this topic is the lack of generalizability of the results because they are often conducted among specific subgroups such as patients with dementia or Alzheimer [1, 16, 17, 24], myocardial infarction [19], or surgery as a result of a hip fracture [25]. Moreover, some of these studies lack power and precision since they are based on small samples [10]. A major methodological shortcoming in some studies is that they fail to take into account death as a competing risk of NHA in the analysis, which may bias the results [1, 12, 18, 26]. Therefore, the objective of this study is to identify predictors of NHA in a Belgian community dwelling population aged 65 years and over [27], considering death as a competing risk factor.

## Methods

### Study population and data

The data for this study were derived from a linkage at the individual level between data from the Belgian health interview survey (BHIS) of 2013 and data from the Belgian compulsory health insurance (BCHI) between 2012 and 2018. This linked data is further referred to as HISlink. The study population is limited to those aged 65 years and older.

The BHIS is a national, cross-sectional household survey conducted more or less every 5 years since 1997 by Sciensano, the Belgian institute for health. Participants are selected from the national population register through a multistage stratified sampling procedure. The participation rate was 57% at household level for the BHIS 2013. In the BHIS, information is collected on the health status, health behavior, health care consumption, use of medicines and sociodemographic characteristics. Post stratification weights were used to obtain representative results at the level of the Belgian population The detailed methodology of the survey is described elsewhere [27].

The BCHI data contain exhaustive and detailed information on the reimbursed health care of over 99% of the total population. These data were provided by IMA, a joint venture of the seven national health insurance organisations that collects and manages all data on healthcare expenditures. The BCHI contains three kinds of data: population data (a limited amount of demographic and socio-economic information), health care expenditure data (information on reimbursed health care) and pharmanet data (detailed information on all prescriptions for reimbursed drugs dispensed in public pharmacies) [6]. Although healthcare consumption is registered in detail, diagnostic information is not available. A proxy for diagnostic information over a number of chronic health conditions (e.g. cardiovascular disorder, diabetes, asthma, epilepsy, chronic obstructive pulmonary disease, Alzheimer’s disease, etc.) is estimated through the volume of the prescription of reimbursed medication using an algorithm defined by a group of experts from the National Institute for Health and Disability Insurance (NIHDI). The algorithm is based on the anatomical, therapeutic, chemical (ATC) codes of specific drugs prescribed and dispensed in public pharmacies. A minimum threshold of 90 DDD (Defined Daily Dose) per year is used. For some chronic conditions, the algorithm takes into account the age of the person to assign the disease or not. For instance, the proxy diagnosis of asthma is more likely to be attributed to people aged 50 years or younger, while those of chronic obstructive pulmonary disease is more likely attributed to people over 50 years to determine cases [28].

Individual BHIS 2013 data were linked with BCHI data using the unique national register number. The linkage rate was 96% for individuals aged 65 years and over. The BHIS sample includes both community dwelling and institutionalized people but for this study we only considered people aged 65 years and more residing at home at the moment of the interview (*n* = 1930).

### Measures

#### Dependent variable

The main outcome was defined as a NHA after participating to the BHIS 2013 at any time during the follow-up period. The BHIS 2013 participants were followed until the date of NHA, the date of death or the end of study period, i.e., December 31, 2018. The information on the date of death was retrieved from the BCHI data. NHA was ascertained if at least one specific nomenclature code defined by the NIHDI for reimbursement purpose and corresponding to care delivery into home for older people or nursing home was found. The date of the first care delivery into NH was considered to be the date of admission into the NH. Only the first admission into NH was considered in this study. Short-stay care episodes, i.e. a maximum stay of 90 days, of which 60 days may be consecutive (defined by specific nomenclature codes) were excluded from the analyses. Therefore, this study focuses on NHA as a permanent resident. NHA for a short stay is not considered.

#### Independent variables

Independent variables were classified according to Andersen’s behavioral model of health care use, as

1. predisposing variables: age, gender, educational attainment, living situation;
2. enabling factors: household income, appreciation of social contacts, home care services use, urbanization level and region of residence;
3. need factors: perceived health, Global Activity Limitations Indicator (GALI), multimorbidity, falls, urinary incontinence or problems in controlling the bladder in the past 12 months (either reimbursement for incontinence protections in BCHI data or self-reported), depression (self-reported depression in the past 12 months or self-reported use of anti-depressants in the past 2 weeks), Alzheimer’s’ disease, number of contacts with health care providers in the past 12 months (general practitioners, specialists, dentists, physiotherapists) and hospitalization in the past 12 months .

Home care services use is based on a single question: “In the last 12 months, have you received help at home or made use of home care services for yourself? (Yes / No)”.This question is preceded by the following intro: “The next question is about home care services that cover a wide range of health and social services provided to people with health problems at their homes. These services comprise for example home care provided by a nurse or midwife, domestic help for older people, “meals on wheels“ or transport service”. Perceived health is based on the single question: “How is your health in general?”. This question is part of the Minimum European Health Module (MEHM), which is internationally used. Five response categories are possible: Very good / Good / Fair / Poor / Very poor. The response categories Very good / Good are recorded as “Good to very Good” and those Fair / Poor / Very poor as “Very bad to fair”.

As multimorbidity indicator we used the number of self-reported chronic conditions per person (out of a total of 25 chronic conditions), in the past 12 months. The list of these 25 chronic conditions is found in Table A1 (supplementary data). The GALI is an indicator of limitations due to health problems taking into account the person’s environment and support. It is based on a single question asking the respondent to estimate the possible restrictions due to their health: “Have you been limited for at least 6 months because of a health problem in the activities that people usually do” (Yes, severely limited / Yes, limited / No, not limited at all) [29]. Information on contact with health care providers and hospitalization were obtained from the BCHI source. Urinary incontinence and Alzheimer’s disease are combined indicators from BCHI and BHIS sources, while the other predictors were based on self-reported information. Alzheimer’s disease cases were ascertained using the aforementioned experts’ algorithm or the use of proxy interview because of a memory problem. The algorithm is based on ATC codes of specific drugs (N06DX01, N06DA) prescribed and dispensed in public pharmacies. The minimum threshold of 90 DDD per year is used to determine cases [28]. Thus, Alzheimer’s disease cases were identified as follows: “use of a minimum of 90 DDD per year of prescribed specific drugs (ATC codes = N06DX01, N06DA)” OR “having had a proxy interview because of a memory problem (e.g. amnesia, senile dementia). Detailed information about the variables description or operationalization are found in Table A1 (supplementary data).

Table 1 provides an overview of the independent variables (levels of variables, data sources and proportion of missing values, if any, before the multiple imputation).

**Table 1:** Overview of the covariates, according to the risk factor groups and data sources, HISlink 2013, Belgium

Covariates	Level of covariates	Data sources		Missing values^**a**^ n (%)
		BHIS	BCHI	
**Predisposing**				
Age		x		–
Gender	Male Female	x		–
Education	Low Middle High	x		19 (0.98)
Living situations	Live alone Not live alone	x		–
**Enabling**				
Household income	Low High	x		280 (14.5)
Level of urbanization	Urban Sub-urban Rural	x		–
Region of residence	Flanders Brussels Wallonia	x		–
Appreciation of social contacts	Rather unsatisfied Rather satisfied	x		440 (22.8)
Home care service use in the past 12 months preceding the survey	Yes No	x		3 (0.16)
**Need**				
Perceived health	Good to very good Very bad to fair	x		426 (22.1)
Multimorbidity		x		18 (0.88)
Long term limitation (GALI)	Yes, severely Yes No	x		452 (23.4)
Falls	Yes No	x		114 (5.91)
Urinary incontinence	Yes No	x	x	5 (0.26)
Depression	Yes No	x		8 (0.41)
Alzheimer’s disease	Yes No	x	x	–
Number of contact with care providers in the past 12 months			x	–
Hospitalization in the past 12 months preceding the survey	Yes No		x	–

HISlink = linkage between BHIS 2013 data and BCHI data from 2012 to 2018

*GALI* Global Activity Limitation Indicator

^a^Missing values before the multiple imputation

### Statistical analyses

#### Descriptive statistics

Baseline characteristics described above were compared by NHA status (admitted to NH or not admitted to NH) with χ2 test for categorical variables, t-test for normally distributed continuous variables and Wilcoxon-Mann-Whitney test for non-normally distributed continuous variables.

Time to NHA was measured in years from the baseline survey to either the date of admission to a NH, date of death (without prior NHA) or end of study period, i.e., December 31, 2018 [20]. Participants who ended their follow-up period were censored [30]. The median follow-up time and median time to NHA and their interquartile range (IQR) were calculated.

#### Competing risk analysis

Competing risks occur frequently in the analysis of survival data [31]. A competing risk is an event whose occurrence precludes the occurrence of the primary event of interest [31–34]. For example, in a study examining time to death attributable to cardiovascular causes, death attributable to non-cardiovascular causes is a competing risk, because subjects who die from another cause are no longer at risk of death due to cardiovascular causes [31, 32, 35]. In the same way, a study of time-to-NHA, death that occurs before NHA is a competing risk as it precludes NHA [36].

In studies involving older people, competing risk of death is especially high due to the higher mortality in this group. Therefore, there is a concern to account for participants who die without experiencing the study outcome of interest. Traditional approaches in survival analysis such as Kaplan-Meier survival analysis and Cox proportional hazards regression are not designed to take into account the competing risk of death [37] and will result in an overestimation of the effect [31, 37]. The higher the death rate among the study population, the more substantial the overestimation.

To account for the presence of competing risks, Fine and Gray [38] proposed to apply the proportional subdistribution hazards model. In this model, estimates are based on modified hazards sets, where subjects experiencing the competing event are retained even after their competing event [33], unlike the Cox model. Whereas the cause-specific hazard function of the Cox model for an event of interest is the instantaneous rate of occurrence of that event in subjects who are currently event-free, the Fine and Gray subdistribution hazard function for a given event is the instantaneous rate of occurrence of that event in subjects who are either currently event-free or who have already experienced a concurrent event [31, 35].

Studies of older individuals in which a substantial number of participants die during follow-up should use the competing risk analysis to accurately determine incidence and effect estimates [36, 37].

Hence, in the current study, a competing risk regression model was used to estimate the association between predictors and NHA, treating death during follow-up as a competing risk.

The cumulative incidence function was used to estimate the risk of NHA over time (using Gray’s method with death as a competing risk [36, 39–41]) for the whole group. Furthermore, as GALI is one of the key indicators in this study due to the impact of limitations on NHA, we also stratified the cumulative incidence function by GALI with the Cuminc R-function [42]. Gray’s test was used to examine for differences between the GALI strata [36, 43].

In addition, we calculated sub-hazard ratios (sHR) of participants’ characteristics for the risk of NHA, with death as a competing risk, also using the Fine and Gray’s proportional sub-hazard model [30, 33, 36, 38, 39, 43].

We imputed missing variables using the fully conditional specification method. The proportion of missing values ranged from 0.16 to 23% (Table 1). As all the variables with missing values are either binary or ordinary, a logistic regression method was used to impute missing values [30, 44]. We created 20 imputed data sets. This number was large enough to achieve a very good efficiency (the relative efficiency was close to 1.0 for all effects). Covariates which were significantly (*P* <  0.05) associated in the univariate analysis (Table A2 in Supplementary data) were retained in the final multivariable model [36]. Several interactions were tested between GALI and the other predictors (age, gender, perceived health and multimorbidity) to account for the possibility that the effect of limitations on the risk of NHA may depend on the level of other predictors. The HAZARDRATIO statement was used in the proportional hazards regression procedure in SAS (PROC PHREG) to produce custom hazard ratios for interactions [45].

For the sensitivity analyses, we used the Cox proportional model on both the imputed and non-imputed data (complete case analysis, *n* = 1209). In the Cox proportional model, participants who died before ever being admitted into a NH and those who ended the study were censored. The results of the sensitivity analyses are reported in the supplementary data.

The assumption of proportional subdistribution hazards was evaluated by including interaction terms between the covariates and time [33, 39]. The assumption was found to be met for all the covariates.

All statistical tests were 2-tailed and we used *p* <  0.05 to determine statistical significance. Analyses were performed using SAS® 9.4 taking into account the complex BHIS design. For the calculation of cumulative incidence function curves we used the package *cmprsk* by Gray [46] of the R project (R version 3.5.2 (2018-12-20).

## Results

### Sample characteristics

The median follow-up time was 5.29 years (95% CI, 5.24–5.32), interquartile range (IQR), 5.12–5.59 years. At the end of follow-up, 226 individuals (13.3%) were admitted to NH with a median time to NHA of 3.0 years, (IQR: 1.5–4.6) and 268 individuals (13.2%) had died before potential NHA. Further information about the sample description (absolute incidences of NHA and death at 1-year follow-up, 3-year follow-up and at the end of study period) can be found in Fig. A1 (supplementary data). Compared to those not admitted to a NH, participants who had been admitted to a NH were at baseline older, female, lower educated, and belonged more often to a lower income household. They also lived more often alone, were rather unsatisfied with their social contacts, had more often experienced falls in the year prior to the survey, and had more often health problems (bad perceived health, limitations, urinary incontinence, cognitive disorders (depression or Alzheimer’s disease), but had less often multimorbidity. Further information on participants’ characteristics can be found in Table 2.

**Table 2:** Baseline characteristics of the study population (*n* = 1930), HISlink 2013, Belgium (weighted)

	Admitted to nursing home ***N*** = 226 (13.3%)	Not admitted to nursing home ***N*** = 1704 (86.7%)	***P***-value
Age, mean (SD)	80.1 (0.59)	73.9 (0.26)	*<.0001*
Gender, n (%)			*0.0192*
Male	106 (45.8)	992 (57.6)	
Female	120 (54.2)	712 (42.4)	
Education, n (%)			*< 0.0001*
Low	144 (68.7)	713 (43.4)	
Middle	46 (17.0)	464 (28.8)	
High	36 (14.3)	527 (27.8)	
Household income, n(%)			*< 0.0001*
Low	180 (82.5)	1106 (66.6)	
High	46 (17.5)	598 (33.4)	
Level of urbanization, n (%)			*0.5857*
Urban	96 (36.9)	823 (42.4)	
Sub-urban	60 (34.2)	405 (30.6)	
Rural	70 (29.0)	476 (27.0)	
Region of residence, n (%)			*0.3470*
Flanders	83 (62.8)	630 (60.7)	
Brussels	37 (5.0)	363 (8.1)	
Wallonia	106 (32.1)	711 (31.2)	
Living situations, n (%)			*<.0001*
Live alone	130 (51.9)	496 (29.0)	
Not live alone	96 (48.1)	1208 (71.0)	
Appreciation of social contacts, n (%)			*0.0175*
Rather unsatisfied	32 (14.7)	132 (8.7)	
Rather satisfied	194 (85.3)	1572 (91.3)	
Home care service use in the past 12 months preceding the survey, n (%)			*<.0001*
Yes	102 (45.3)	346 (19.6)	
No	124 (54.7)	1358 (80.4)	
Perceived health, n (%)			*0.0049*
Good to very good	111 (50.4)	1113 (66.8)	
Very bad to fair	115 (49.6)	591 (33.2)	
Multimorbidity, median (Q1-Q3)^a^	1.64 (0.5–3.5)	1.79 (0.6–2.9)	*0.0067*
Long term limitation (GALI), n (%)			*0.0088*
Yes, severely	30 (16.1)	160 (10.2)	
Yes	87 (32.9)	477 (25.5)	
No	99 (51.0)	1067 (64.3)	
Falls, n (%)			*<.0001*
Yes	79 (36.2)	286 (16.9)	
No	147 (63.8)	1418 (83.1)	
Urinary incontinence, n (%)			*<.0001*
Yes	49 (22.8)	176 (10.7)	
No	177 (77.2)	1528 (89.3)	
Depression, n (%)			*<.0001*
Yes	50 (24.7)	195 (12.1)	
No	176 (75.3)	1509 (87.9)	
Alzheimer’s disease, n (%)			*<.0001*
Yes	13 (12.6)	35 (1.7)	
No	213 (87.4)	1669 (98.3)	
Number of contact with care providers in the past 12 months, median (Q1-Q3)^a^	12.66 (7.9–18.7)	11.2 (6.3–17.7)	*0.0002*
Hospitalization in the past 12 months preceding the survey, n (%)			*0.0380*
Yes	59 (28.2)	323 (19.4)	
No	167 (71.8)	1381 (80.6)	

HISlinkk = linkage between BHIS 2013 data and BCHI data from 2012 to 2018

*Q1* lower quartile, *Q3* upper quartile, *GALI* Global Activity Limitation Indicator

^a^Non-parametric test (Wilcoxon Two-Sample Test)

### Cumulative risk of nursing home admission

**Fig. 1:**
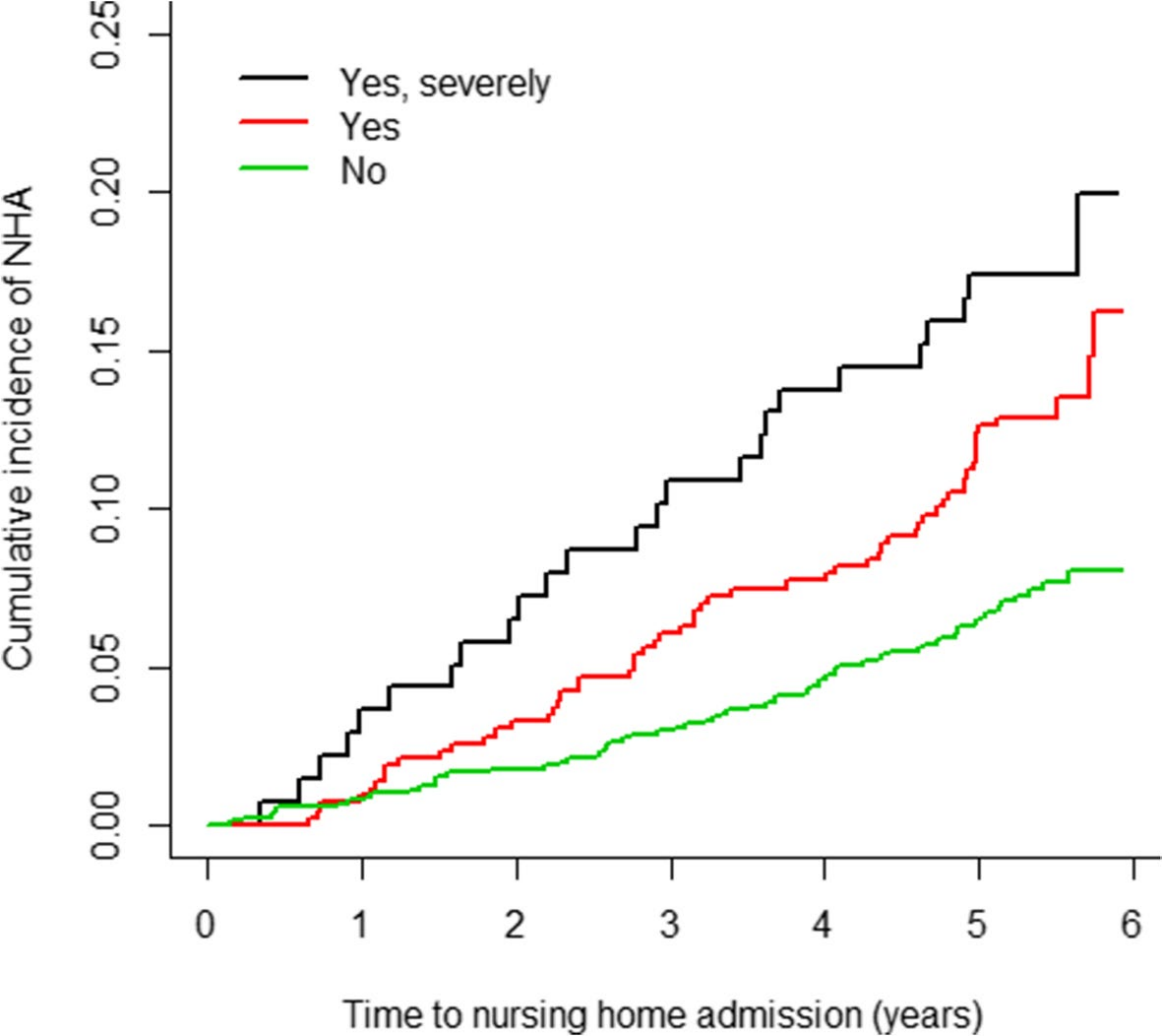
Crude cumulative risk of nursing home admission, stratified by limitations (GALI), accounting death as a competing risk, HISlink 2013, Belgium

The overall crude cumulative risk of NHA was 1.4% (95% CI, 0.9–2.1) at 1 year follow-up, 5.7% (95% CI, 4.7–6.8) at 3 years follow-up and 13.1% (95% CI, 11.3–15.0) at the end of follow-up (5.9 years). Figure 1 provides the unadjusted cumulative incidence curves for NHA, stratified by GALI, with death as a competing risk. The crude risk of NHA was significantly different in function of the severity of limitations (GALI) at least in one category as compared to the other categories (Gray’s test: χ2 = 22.28, *p* <  0.0001). Individuals who were severely limited had a higher risk of NHA at any time point of follow-up than those who were not. For instance, at the end of the study, the risk of NHA was significantly higher among individuals with severe limitations (20.0% (95% CI, 12.7–28.5)) than those without limitations (8.0% (95% CI, 6.2–10.1)). Whereas the risk of NHA was not statistically different between individuals with severe limitations (20.0% (95% CI, 12.7–28.5) as compared to those with moderate limitations (16.2% (95% CI, 11.6–21.5)), (Fig. 1).

### Results of the subdistribution hazard model for nursing home admission

The results of the multivariable competing risk analysis are displayed in Table 3.

**Table 3:** Predictors for nursing home admission: results from the competing risk regression (*N* = 1930), HISlink 2013, Belgium

Potential predictors	sHR (95% CI)
**Predisposing**	
Age	1.09 (1.08–1.10)**
Female	0.75 (0.70–0.79)**
Educational attainment (Ref. = High)	
Low	1.44 (1.26–1.65)**
Middle	0.86 (0.75–0.98)*
Living alone	1.68 (1.57–1.80)**
**Enabling**	
Low household income	1.22 (1.00–1.50)
Unsatisfied with the social contacts	1.31 (0.73–2.37)
Home care service use in the past 12 months preceding the survey (Ref. =No)	1.57 (1.48–1.68)**
**Need**	
Long term limitation (GALI) and perceived health interaction^a^	
Bad perceived health vs. good perceived health at severe limitations	0.30 (0.29–0.31)*
Bad perceived health vs. good perceived health at moderate limitations	0.97 (0.95–0.98)*
Bad perceived health vs. good perceived health at no limitations	1.89 (1.85–1.92)*
Severe limitations vs. no limitations at good perceived health	2.42 (2.35–2.49)*
Severe limitations vs. no limitations at bad perceived health	0.41 (0.40–0.42)*
Severe limitations vs. moderate limitations at good perceived health	2.09 (2.03–2.16)*
Severe limitations vs. moderate limitations at bad perceived health	0.65 (0.64–0.66)*
Moderate limitations vs. no limitations at good perceived health	1.22 (1.20–1.24)*
Moderate limitations vs. no limitations at bad perceived health	0.64 (0.63–0.66)*
Multimorbidity	0.94 (0.90–0.97)*
Falls (Ref. = No)	1.76 (1.64–1.89)**
Urinary incontinence (Ref. = No)	1.48 (1.22–1.79)*
Depression (Ref. = No)	1.45 (1.25–1.70)**
Alzheimer disease (Ref. = No)	3.47 (3.05–3.96)**
Number of contact with health care providers in the past 12 months preceding the survey	0.98 (0.97–0.99)**
Hospitalization in the past 12 months preceding the survey (Ref. = No)	1.07 (0.95–1.21)

HISlink = linkage between BHIS 2013 data and BCHI data from 2012 to 2018

^a^The HAZARDRATIO statement was used in PROC PHREG to produce custom hazard ratios for interactions

*NHA* nursing home admission, *sHR* sub Hazard Ratios, *GALI* Global Activity Limitation Indicator

To facilitate reading, the GALI categories were reported as severe limitations, moderate limitations and no limitations, which referred to the categories yes, severely limited, yes limited and no, not limitation at all respectively; * *p* < 0.05; ** *p* < 0.0001

Among the predisposing factors, a 1 year increase in age is associated with a 9% increase risk of NHA. Individuals with lower educational attainment also showed a higher risk of NHA (sHR = 1.44, 95% CI, 1.26–1.65) as compared to those with higher educational attainment, as well as living alone (sHR = 1.68, 95% CI, 1.57–1.80). Being female, having an intermediate educational attainment were associated with a 25% and 14% reduction in the risk of NHA respectively.

Regarding the enabling factors, use of home care services in the past 12 months (sHR = 1.57, 95% CI, 1.48–1.68) were associated with a higher risk of NHA.

With respect to the need factors, the interaction between limitations and perceived health was found to be statistically significant and interaction terms were therefore added in the final model. Because of this interaction, the interpretation of the sHRs of perceived health must take into account the levels of limitations and vice versa. Our results show for the impact of limitations, that 1) if people are in good perceived health, severe limitations (sHR = 2.42, 95% CI, 2.35–2.49) increase the risk on NHA; and 2) if people are in bad perceived health, severe limitations (sHR = 0.41, 95% CI, 0.40–0.42) decrease the risk of NHA. For the impact of perceived health, 1) if people have no limitations, bad perceived health increases the risk of NHA substantially (sHR = 1.89, 95% CI, 1.85–1.92); and 2) if people have severe limitations, bad perceived health decreases the risk of NHA (sHR = 0.30, 95% CI, 0.29–0.31). Further information about the results on interactions can be found in Table 3.

Individuals experiencing falls in the past 12 months (sHR = 1.76, 95% CI, 1.64–1.89), those suffering from urinary incontinence (sHR = 1.48, 95% CI, 1.22–1.79), and those suffering from depression (sHR = 1.45, 95% CI, 1.25–1.70) had a higher risk of NHA than their counterparts. A one unit increase in the mean number of chronic conditions (multimorbidity) resulted in a 6% smaller risk of NHA (sHR = 0.94, 95% CI, 0.90–0.97). An increased number of contacts with health care providers was associated with a decreased risk of NHA. Suffering from Alzheimer is the strongest predictor of NHA, with a sHR of 3.47 (95% CI, 3.05–3.96).

## Discussion

### Summary of the results

To our knowledge this is the first study that investigated predictors of NHA among the Belgian community dwelling population aged 65 years and older.

The overall unadjusted cumulative incidence (risk) of NHA, accounting for death as competing risk, was of 5.7% at 3 years of follow-up and of 13.1% at the end of the study. After adjusting for baseline characteristics of participants, important predictors of NHA were found. These were, among others, being older, living alone, having used of home care services, having a history of falls, depression or Alzheimer’s disease, all of which were significantly associated with a higher risk of NHA. Predictors such as being female, having multimorbidity and increased contacts with health care providers were significantly associated with a decreased risk of NHA.

### Incidence of NHA

The incidence of NHA may be influenced by organizational aspects and cultural aspects [47], but also by the availability, accessibility and affordability of home care facilities. The characteristics of the study population also play a role since the cumulative incidence of NHA could be affected by the higher risk of death in the population under study. Previous studies have investigated predictors of NHA among sub-groups of the population. For instance, Bergkamp et al. [36] investigated predictors of NHA in Cerebral Small Vessel Disease (CSVD) patients and have found that after 5-years follow-up, the cumulative incidence was 3.6% (95% CI, 2.2–5.5) and 6% (95% CI, 4.2–8.3) after 8 years of follow-up. This cumulative incidence is lower than those found in our study. A possible explanation could be that the risk of the competing event, i.e., death, is likely to be higher in patients suffering from CSVD than in the general population, which may affect the risk of NHA. In contrast, our cumulative incidence is lower than the cumulative incidence found by Wolff et al. in community living older adults in the USA (16.1% in a 2-years follow-up) [48]. This difference could be explained by differences in the characteristics of the study population. Indeed, the Wolff et al. study participants were much older (sample mean age of 79 years compared to 74.7 years in our study), received assistance with personal care or mobility from a family member or unpaid caregiver (help with 2 of 6 self-care activities) and nearly 1 in 3 had dementia.

### Factors associated with NHA

Important predictors of NHA were identified in our study. This will be discussed in the following paragraphs in relation to findings from previous studies and according to the Andersen behavioural model.

In earlier studies, advanced age emerged as a strong predictor of NHA among the predisposing factors [12, 25, 26]. In accordance with these studies, higher age was also found to be a significant predictor of NHA, even after taking into account the competing risk of death. With regard to gender, women were found to be less likely to enter NH than men. This result is consistent with the study by Gaugler et al. [26] (female: HR of risk: 0.87 (95% CI, 0.81–0.93)). Although this is an unexpected result compared to the results presented in Table 2 and the univariate analysis (Table A2, supplementary data), it could be explained by a protective effect of the female sex against NHA compared to men. Previous studies have also found a protective effect of female gender against NHA [25, 26, 49]. Casanova et al. (2021) argue that the protective effect of female gender against NHA may be explained by a stronger negative preference for NH care among women or by the fact that children provide more informal care for women than for men [49]. Living situations appeared as the strongest predictor of NHA among the predisposing factors. Individuals who lived alone had nearly twice the sHR to enter a NH. Our findings are in line with those in previous studies [20, 50]. Although older people prefer living in their own home as long as possible [7, 11, 51] this may be more difficult for people living alone because of lack of social support and lack of informal care.

Among the enabling factors, the use of home care services in the previous year was associated with a greater risk of NHA. This result is not surprising since the use of home care services is generally an expression of a need for support and therefore a first step towards possible NHA. Our result is similar to those in a study on predictors of NHA after hip fracture. The authors found that receiving home care before injury was associated with an increase in HR of 2.00 (95% CI 1.54–2.61), HR 1.64 (95% CI, 1.43–1.87), and HR 1.22 [95% CI, 1.13–1.32) for patients aged 60 to 69 years, 70 to 79 years, and 80 to 89 years respectively [25].

Within the need factors, if either poor perceived health or severe limitations are present there is an increased risk of NHA, but when they occur together the risk of NHA decreases, most likely because for those people the risk of dying is larger than the risk of being admitted to a NH (competing risk). For instance, the risk of dying in case of bad perceived health and severe limitations at 1, 3 and 5-years follow-up is 8.5, 23.9 and 35% respectively. The risk of NHA in case of bad perceived health and severe limitations at the same time points is 2.6, 9.4 and 16.5% respectively (Table A5, supplementary data). The paradoxical finding of a decreasing risk of NHA when both poor perceived health and severe limitations are present is in line with the fact that an increasing number of chronic diseases was associated with a reduced risk of NHA, probably because of competing risk of death among this group. Indeed, the higher the number of chronic conditions the higher the risk of poor perceived health and more severe limitations.

In line with earlier studies [26, 52], we found that a history of falls in the past 12 months was associated with an increased risk of NHA. In fact, in some cases, falls among the older people can lead to more serious events (fractures, injuries, loss of autonomy) with adverse consequences on their health status and therefore precipitate their admission to a NH.

The presence of Alzheimer’s disease is by far the strongest predictor of NHA. In the literature, beside age, cognitive comorbidities (depression, Parkinson, dementia or Alzheimer’s disease) and functional impairment were among the strongest predictors and are associated with an increased risk of NHA [12, 15, 25, 26, 53]. For example, in a study among a general older population, the authors found that Alzheimer’s or dementia increases the hazard of NHA by 20.2 times for men and 10.0 times for women [12]. In another study of 137,000 community dwelling patients aged 65 years or more, Harris et al. found that depression was associated with a higher risk of NHA in the general population [53]. Another interesting but surprising result is that with an increasing number of contacts with health care providers, the risk of NHA decreased. This finding could be explained on the one hand by the fact that people with a higher number of contacts are likely to be in poorer health and therefore less likely to enter a nursing home due to a higher risk of death. On the other hand, an increased number of contacts with health care providers will allow appropriate treatment and therefore prevent or delay NHA. Luppa et al. (2010) [18] also found a decreased risk of NHA with an increased number of specialist visits and explained this finding as a positive effect of appropriate treatment of medical conditions by specialists. Other need factors are of lesser importance.

### Strengths and limitations

From a public health perspective, the major strengths of this study include the use of a large sample and the use of a large number of individual-level predictors, a relatively long follow-up period, and the linkage to administrative data to identify NHA and/or death. The use of the competing risk analysis is another strength of this study. Indeed, we performed competing risk regression to study the association between several covariates and the risk of NHA. This approach is preferred over a standard survival model because in older population, death may compete with NHA, and ignoring such competing risk may lead to biased results [37]. In competing risk situations, the cumulative incidence function was more appropriate as it took competing events into account when estimating the incidence. We further chose the Fine and Gray model over the cause-specific hazard model as our primary interest was in predictive modelling.

The current study has some limitations that deserves to be pointed out. First, the exact dates of NHA were not available. We used the dates of the first registered care in a NH based on specific nomenclature code as a proxy of dates of NHA. However, these dates are quite accurate and deviations from the exact dates are small. Second, almost all covariates included in the analysis were measured at baseline and most of them are self-reported. Therefore, possible changes (e.g., in living situations or social support) over the course of the study are not taken into account and the risk of reporting bias remains. Third, although in recent years efforts have been made to avoid NHA by taking measures to facilitate home health care, our data did not allow demonstrating this. To investigate this thoroughly, longitudinal data are required on both the evolution of the health situation and the use of home health care, but unfortunately such information was not available in our study. Fourth, data on local variations in supply of care and/or home care services (supply of NH beds, hospital beds, and physicians in the region of residence, waiting lists, etc.) as potential important confounders at the enabling level were unavailable and therefore not included in our analyses. Fifth, due to the lack of diagnostic information, Alzheimer’s disease indicator was estimated based on prescribed specific medications and self-reported information on memory problem (e.g. amnesia, senile dementia), making it less sensitive. Indeed, many people with Alzheimer do not take specific medications. So individuals suffering from this disease might be underestimated. Sixth, although this study was conducted in a large dataset, a selection bias is unavoidable and the representativeness of the sample is not guaranteed. However, through the calculation of post stratification weights, with the Belgian national registry as auxiliary data source, results are as representative as possible of the Belgian community dwelling population. Finally, the finding of this study may not be generalized to other areas or settings with lower health system standards, for example because the organization of the Belgian health care system can be very different from other countries.

### Implications and challenges for the future

This study has implications for practitioners and policy makers. As a result of the ageing population the pressure on NH will only increase. Efforts and measures that enable older people to remain longer at home will not only be beneficial from a budgetary point of view but will also increase the wellbeing of older people. This study identified some domains in which health care professionals and policy makers should further invest to delay NHA. Prevention of falls is a first important point. Home care givers should also be trained to deal with mental health problems. Attention should also be paid to the problems of urinary incontinence of older people. Adaptation of the home environment in a way that despite their limitations, older people can still continue their daily activities is of utmost importance. Finally, the strong association found between Alzheimer’s disease and NHA is of course not surprising. Alzheimer’s disease, dementia and severe cognitive problems are important reasons why people have to be admitted in a NH. Therefore, at population level, further efforts are needed to prevent important risk factors for dementia. Focus should also be on people living alone to provide the appropriate social support and more timely contacts with health care providers. Further investigation of predictors of NHA should include contextual enabling actors such as the supply of nursing-home beds, hospital beds, physicians and waiting lists for NHA. Analysis taking into account other competing events such as home health care services should also be considered.

## Conclusions

Our findings underline important predictors of NHA of older adults, and therefore offer possibilities of prevention to avoid or delay NHA for this population. Practical implications include prevention of falls, management of people with urinary incontinence at home, and appropriate and timely management of limitations, depression and Alzheimer’s disease. Focus should also be on people living alone to provide more timely contacts with health care providers. Further investigation of predictors of NHA should include contextual enabling factors such as the supply of nursing-home beds, hospital beds, physicians and waiting lists for NHA.

## Supplementary Information

**Additional file 1.**

## Data Availability

The survey datasets and linked health administrative data analyzed in the current study are not publicly available due to restrictions based in the General Data Protection Regulation (GDPR) on sensitive data such as personal health data. BHIS data contains sensitive and identifying information and therefore must only be made available upon request. Requests for data access may be made to the Social Security and Health Chamber of the Information Security Committee (hereinafter referred to as the “Social Security and Health Chamber”). Further information regarding the survey and the data access procedure can be found here: https://www.sciensano.be/en/node/55737/health-interview-survey-microdata-request-procedure.

## Abbreviations

ATC: Anatomical Therapeutic Chemical
BCHI: Belgian compulsory health insurance
BHIS: Belgian health interview survey
CSVD: Cerebral Small Vessel Disease
DDD: Daily defined dose
GALI: Global Activity Limitations Indicator
HR: Hazard Ratio
HISlink: linkage between BHIS data and BCHI data
IEA: International Epidemiological Association
IMA: Inter Mutualistic Agency
LTC: Long-term care
MEHM: Minimum European Health Module
NH: Nursing home
NHA: Nursing Home Admission
NIHDI: National Institute for Health and Disability Insurance
SHR: Sub Hazard Ratio
WHO: World Health Organization
