# Supplementary material for: Predictors of nursing home admission in the older population in Belgium: a longitudinal follow-up of health interview survey participants
Source: BMC Geriatr. 2022 Oct 20;22:807. doi: 10.1186/s12877-022-03496-4 (PMC9585772; doi:10.1186/s12877-022-03496-4)
Supplement: Supplementary file 1 — Additional file 1. [file 12877_2022_3496_MOESM1_ESM.docx]

**Supplementary data**

Table A1: Variables description / operationalization

| Variables name | Variable description / operationalization |
| --- | --- |
| Age | Respondents age (in years) |
| Gender | Respondents gender (Male / Female) |
| Educational attainment | Educational attainment was based on the highest level of education achieved according to the ISCED 1997 and recoded unto three categories: low (lower secondary education or less), intermediate (higher secondary education), and high (higher education). |
| Living situations | This variable is based on number of household members, recorded as “living alone” if one household member and “living with someone” otherwise. |
| Household income | The equivalised household income quintiles were recorded in low (quintile 1-3) and high (quintile 4 and 5). |
| Appreciation of social contacts | This variable is based on the response to the question: “How do you find your social contacts? (Very satisfied / Somewhat satisfied / Somewhat  unsatisfied / Really unsatisfied)”. The response categories Very satisfied / Somewhat satisfied are recorded as “Rather satisfied” and those Somewhat satisfied / Somewhat unsatisfied are recorded as “Rather unsatisfied”. |
| Home care service use in the past 12 months preceding the survey | The question on home care service use is preceded with an intro: “The next question is about home care services that cover a wide range of health and social services provided to people with health problems at their homes. These services comprise for example home care services provided by a nurse or midwife, home help for the housework or for older people, "meals on wheels" or transport service: “In the last 12 months, have you received help at home or made use of home care services for yourself? (Yes / No)”. |
| Level of urbanization | The indicator level of urbanization are created using the municipality of residence of the respondents (based on information in the National Register). Three levels of urbanization were defined: suburban, urban and rural. |
| Region of residence | This variable is based on the region of residence of the respondents: Flanders, Brussels and Wallonia. |
| Perceived health | This self-reported indicator is based on the question: “How is your health in general?”. Five response categories are possible: Very good / Good / Fair / Poor / Very poor. The response categories Very good / Good are recorded as “Good to very  Good” and those Fair / Poor / Very poor as “Very bad to fair”. |
| Long term limitation (GALI) | The GALI is an indicator of limitations due to health problems taking into account the person’s environment and support. It is based on a single question asking the respondent to estimate the possible restrictions due to their health: “Have you been limited for at least 6 months because of a health problem in the activities that people usually do” (Yes, severely limited / Yes, limited / No, not limited at all). |
| Multimorbidity | As multimorbidity indicator we used the number of self-reported chronic conditions per person (out of a total of 25 chronic conditions), in the past 12 months preceding the survey. The 25 chronic conditions taken into account are: asthma; glaucoma; chronic bronchitis, chronic obstructive pulmonary diesease or emphysema; cataract; serious heart disease; Parkinson's disease; high blood pressure; epilepsy; stroke (or consequences); chronic fatigue for a period of at least 3 months; rheumatoid arthritis or osteoarthritis; osteoporosis; diabetes; disorder of the larger or the small bowel for at least 3 months; allergy; serious disease of the kidney or stones in the kidney; stomach ulcer; chronic cystitis; cirrhosis of the liver, liver dysfunction; serious or chronic skin disease; cancer; gallstones of inflammation of the gallbladder; severe headache such as migraine; serious gloom or depression; thyroid problems. |
| Falls | This variable is based on a single question: “In the past 12 months, have you had any fall including a slip or trip in which you lost your balance and landed on the floor or ground or lower level?” (Yes / No). |
| Urinary incontinence | This variable is based on whether respondents had a package for incontinence in the Belgian Compulsory Health Insurance (BCHI) data in the past 12 months (with specific nomenclature codes) or whether they reported urinary incontinence or bladder control problems in the last 12 months: "In the past 12 months, have you had urinary incontinence, bladder control problems" (Yes / No) |
| Depression | This variable is based on whether the respondent had self-reported depression in the past 12 months: “In the past 12 months, have you had serious gloom or depression for a period of at least 2 weeks?” (Yes / No) or whether the respondent self-reported use of prescribed antidepressants in the past 2 weeks: “During the past 2 weeks, have you used any antidepressants that were prescribed for you by a doctor?” (Yes / No). |
| Alzheimer’s disease | This variable is based on whether the respondent had used a minimum of 90 Daily Defined Dose (DDD) per year of prescribed specific drugs (ATC codes = N06DX01, N06DA)" or "have had a proxy interview because of a memory problem (e.g. amnesia, senile dementia). |
| Number of contact with health care providers in the past 12 months preceding the survey | Number of contact with general practitioners, specialists, dentists, physiotherapists in the past 12 months preceding the survey, based on specific nomenclature codes in the BCHI administrative data. |
| Hospitalization in the past 12 months preceding the survey | Having had inpatient hospitalization in the past 12 months preceding the survey in the BCHI administrative data. |


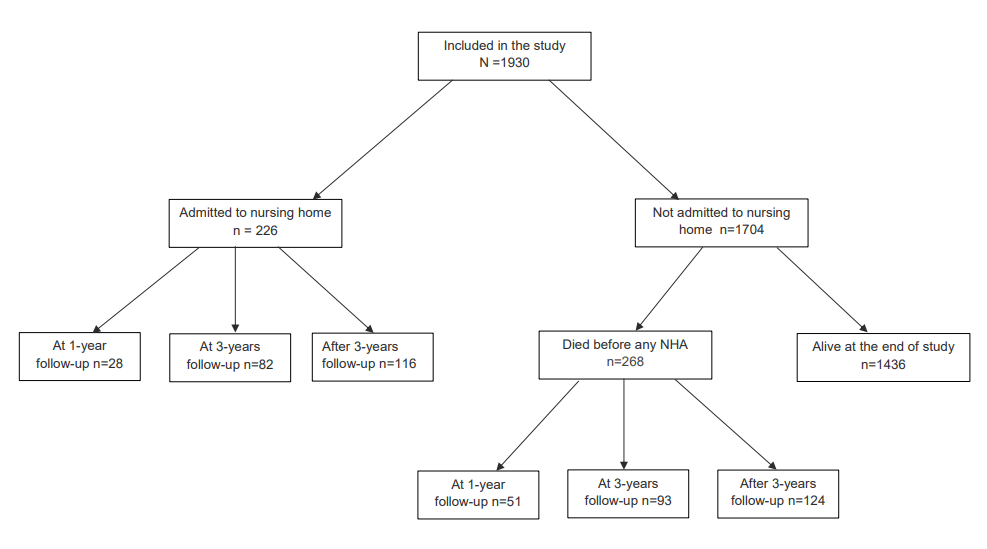


Figure A1: Sample description at 1 year, 3 years and the end of follow-up period

Table A2: Predictors for nursing home admission: results from univariate competing risks analysis (N=1930), HISlink 2013, Belgium

| Potential predictors | **sHR**  **(95% CI)** |
| --- | --- |
| **Predisposing** |  |
| Age | 1.10 (1.09-1.12)** |
| Female | 1.54 (1.53-1.55)** |
| Educational attainment (Ref. = High) |  |
| Low | 2.71 (2.58-2.86)** |
| Middle | 1.03 (0.97-1.09) |
| Living alone | 2.46 (2.44-2.48)** |
| **Enabling** |  |
| Low household income | 2.26 (2.07-2.47)** |
| Unsatisfied with the social contacts | 1.64 (1.16-2.32)* |
| Home care service use in the past 12 months preceding the survey (Ref. =No ) | 3.26 (3.23-3.30)** |
| Level of urbanization (Ref. =Rural) |  |
| Urban | 0.81 (0.49-1.34) |
| Sub-urban | 0.99 (0.59-1.69) |
| Region of residence (Ref. = Flanders) |  |
| Brussels | 0.62 (0.39-1.00) |
| Wallonia | 1.00 (0.68-1.48) |
| **Need** |  |
| Bad perceived health (Ref. = Good perceived health) | 1.92 (1.54-2.39)** |
| Long term limitation (GALI) (Ref. = no limitations) |  |
| Severe limitations | 1.87 (1.42-2.46)* |
| Moderate limitations | 1.58 (1.29-1.93)* |
| Multimorbidity | 1.09 (1.08-1.10)** |
| Falls (Ref. = No) | 2.64 (1.54-2.74)** |
| Urinary incontinence (Ref. = No) | 2.41 (2.37-2.44)** |
| Depression (Ref. = No) | 2.12 (2.09-2.15)** |
| Alzheimer disease (Ref. = No) | 6.63 (6.54-6.71)** |
| Number of contact with health care providers in the past 12 months preceding the survey | 1.02 (1.01-1.05)** |
| Hospitalization in the past 12 months preceding the survey (Ref. = No) | 1.54 (1.53-1.56)** |

*HISlink = linkage between Belgian Health Interview Survey (BHIS) 2013 data and Belgian Compulsory Health Insurance (BCHI) data from 2012 to 2018; sHR = sub Hazard Ratios; GALI = Global Activity Limitation Indicator; To facilitate reading, the GALI categories were reported as severe limitations, moderate limitations and no limitations, which referred to the categories yes, severely limited, yes limited and no, not limitation at all respectively; * p < 0.05; ** p < 0.0001.*

**Sensitivity analysis**

Sensitivity analysis was performed by repeating the analysis using a Cox proportional hazards regression (Table A3) and a competing risk analysis without imputation of missing values, i.e., a complete case analysis, n = 1209 (Table A4). Compared to the results from the competing risk analysis as presented in Table 3, we found that results from applying Cox model are overestimated in a large set of the covariates as expected (e.g., for living alone: sHR = 1.73 for Cox model vs. 1.68 for competing risk analysis; Alzheimer’s disease: sHR = 3.60 for Cox model vs. 3.47 for competing risk analysis) and unlike the findings from the competing risk analysis, some associations were not significant (e.g., gender, educational attainment, multimorbidity, urinary incontinence, etc.). We believe that these inconsistencies do not affect the conclusions of this study. The non-significant effects observed in the Cox model seem to be related to the sample size behind the two approaches (in the competing analysis, subjects experiencing the competing event are retained even after their competing event, unlike the Cox model) rather than to other reasons. For example, for urinary incontinence, the sHR from the competing risks analysis is 1.48 (95% CI: 1.22-1.79) and the HR from the Cox model is 1.49 (95% CI: 0.92-2.42), which are almost similar in terms of direction and magnitude of effect.

Regarding the results from the complete case analysis, although the magnitude of the associations is slightly different as compared to those after imputation, the direction of the effects and conclusions remain, in overall terms unchanged.

Table A3: Predictors for nursing home admission: results from Cox proportional hazards regression (N=1930), HISlink 2013, Belgium

| Potential predictors | **HR**  **(95% CI)** |
| --- | --- |
| **Predisposing** |  |
| Age | 1.09 (1.06-1.13)** |
| Female | 0.69 (0.46-1.06) |
| Educational attainment (Ref. = High) |  |
| Low | 1.46 (0.73-2.91) |
| Middle | 0.92 (0.44-1.89) |
| Living alone | 1.73 (1.08-2.77)* |
| **Enabling** |  |
| Low household income | 1.19 (0.64-2.22) |
| Unsatisfied with the social contacts | 1.32 (0.53-3.26) |
| Home care service use in the past 12 months preceding the survey (Ref. =No ) | 1.64 (1.03-2.61)* |
| **Need** |  |
| Long term limitation (GALI) and perceived health interaction^a^ |  |
| Bad perceived health vs. good perceived health at severe limitations | 0.43 (0.13-1.75) |
| Bad perceived health vs. good perceived health at moderate limitations | 1.11 (0.54-2.27) |
| Bad perceived health vs. good perceived health at no limitations | 2.24 (1.27-3.97)* |
| Severe limitations vs. no limitations at good perceived health | 2.66 (1.00-7.28) |
| Severe limitations vs. no limitations at bad perceived health | 0.42 (0.18-0.97) |
| Severe limitations vs. moderate limitations at good perceived health | 2.42 (0.83-7.19) |
| Severe limitations vs. moderate limitations at bad perceived health | 0.79 (0.37-1.70) |
| Moderate limitations vs. no limitations at good perceived health | 1.16 (0.63-2.14) |
| Moderate limitations vs. no limitations at bad perceived health | 0.56 (0.28-1.08) |
| Multimorbidity | 0.91 (0.81-1.02) |
| Falls (Ref. = No) | 1.81 (1.15-2.84)* |
| Urinary incontinence (Ref. = No) | 1.42 (0.81-2.52) |
| Depression (Ref. = No) | 1.69 (0.95-2.99) |
| Alzheimer disease (Ref. = No) | 3.60 (1.37-9.48)* |
| Number of contact with health care providers in the past 12 months preceding the survey | 0.99 (0.98-1.00) |
| Hospitalization in the past 12 months preceding the survey (Ref. = No) | 1.42 (0.87-2.31) |

*HISlink = linkage between BHIS 2013 data and BCHI data from 2012 to 2018; ^a^ The HAZARDRATIO statement was used in PROC* PHREG *to produce custom hazard ratios for interactions; HR = Hazard Ratios; GALI = Global Activity Limitation Indicator; To facilitate reading, the GALI categories were reported as severe limitations, moderate limitations and no limitations, which referred to the categories yes, severely limited, yes limited and no, not limitation at all respectively; * p < 0.05; ** p < 0.0001.*

Table A4: Predictors for nursing home admission: results from competing risks analysis of complete case analysis (N=1209), HISlink 2013, Belgium

| Potential predictors | **sHR**  **(95% CI)** |
| --- | --- |
| **Predisposing** |  |
| Age | 1.08 (1.09-1.10)** |
| Female | 1.00 (0.99-1.02) |
| Educational attainment (Ref. = High) |  |
| Low | 1.77 (1.74-1.81)** |
| Middle | 0.85 (0.83-0.87)** |
| Living alone | 1.39 (1.37-1.41)** |
| **Enabling** |  |
| Low household income | 1.61 (1.58-1.64)** |
| Unsatisfied with the social contacts | 1.93 (1.90-1.97)** |
| Home care service use in the past 12 months preceding the survey (Ref. =No ) | 1.26 (1.24-1.27)** |
| **Need** |  |
| Long term limitation (GALI) and perceived health interaction^a^ |  |
| Bad perceived health vs. good perceived health at severe limitations | 0.14 (0.13-0.15)* |
| Bad perceived health vs. good perceived health at moderate limitations | 0.97 (0.91-0.95)* |
| Bad perceived health vs. good perceived health at no limitations | 1.93 (1.90-1.96)* |
| Severe limitations vs. no limitations at good perceived health | 4.98 (4.79-5.19)* |
| Severe limitations vs. no limitations at bad perceived health | 0.37 (0.36-0.38)* |
| Severe limitations vs. moderate limitations at good perceived health | 4.22 (4.05-4.40)* |
| Severe limitations vs. moderate limitations at bad perceived health | 0.65 (0.63-0.66)* |
| Moderate limitations vs. no limitations at good perceived health | 1.18 (1.16-1.20)* |
| Moderate limitations vs. no limitations at bad perceived health | 0.57 (0.56-0.58)* |
| Multimorbidity | 0.90 (0.88-0.91)** |
| Falls (Ref. = No) | 1.38 (1.37-1.40)** |
| Urinary incontinence (Ref. = No) | 1.88 (1.86-1.91)** |
| Depression (Ref. = No) | 1.62 (1.60-1.65)** |
| Alzheimer disease (Ref. = No) | 4.99 (4.88-5.10)** |
| Number of contact with health care providers in the past 12 months preceding the survey | 0.98 (0.97-0.99)** |
| Hospitalization in the past 12 months preceding the survey (Ref. = No) | 1.42 (1.40-1.44)** |

*HISlink = linkage between BHIS 2013 data and BCHI data from 2012 to 2018; ^a^ The HAZARDRATIO statement was used in PROC* PHREG *to produce custom hazard ratios for interactions; sHR = sub Hazard Ratios; GALI = Global Activity Limitation Indicator. To facilitate reading,, the GALI categories were reported as severe limitations, moderate limitations and no limitations, which referred to the categories yes, severely limited, yes limited and no, not limitation at all respectively; * p < 0.05; ** p < 0.0001.*

Table A5: Risk of dying and risk of NHA by level of limitations and by perceived health at 1, 3 and 5-years follow-up

| GALI | Bad perceived health | | | | | | Good perceived health | | | | | |
| --- | --- | --- | --- | --- | --- | --- | --- | --- | --- | --- | --- | --- |
|  | % Risk of death (95% CI) | | | % Risk of NHA (95% CI) | | | % Risk of death (95% CI) | | | % Risk of NHA (95% CI) | | |
|  | At 1-year | At 3-year | At 5-years | At 1-year | At 3-year | At 5-years | At 1-year | At 3-year | At 5-years | At 1-year | At 3-year | At 5-years |
| Severe limitations | 8.5 (4.4-14.5) | 23.9 (16.6-32.0) | 35.0 (26.5-43.7) | 2.6 (0.7-6.7) | 9.4 (5.0-15.5) | 16.2 (10.2-23.5) | 0 | 10.0 (1.6-2.8) | 20.0 (5.9-40.0) | 10.0 (1.6-27.8) | 20.0 (6.0-39.9) | 25.0 (8.7-45.5) |
| Moderate limitations | 2.7 (1.2-5.8) | 11.1 (7.7-15.3) | 17.7 (13.3-22.6) | 1.1 (0.3-3.1) | 6.1 (3.7-9.5) | 12.7 (9.0-17.1) | 1.2 (0.2-3.9) | 2.4 (0.8-5.7) | 6.0 (3.1-10.4) | 0.6 (0.05-3.1) | 6.0 (1.8-3.1) | 12.1 (7.6-17.5) |
| No limitations | 1.4 (0.3-4.5) | 5.5 (2.6-10.1) | 11.0 (6.6-16.8) | 1.4 (0.3-4.5) | 5.5 (2.6-10.1) | 13.1 (8.2-19.2) | 0.9 (0.4-1.8) | 3.8 (2.6-5.3) | 7.8 (6.1-9.9) | 0.6 (0.2-1.5) | 2.3 (1.4-3.6) | 5.1 (3.7-6.8) |
